# Changes in the Composition of the Gut Microbiota and the Blood Transcriptome in Preterm Infants at Less than 29 Weeks Gestation Diagnosed with Bronchopulmonary Dysplasia

**DOI:** 10.1128/mSystems.00484-19

**Published:** 2019-10-29

**Authors:** Feargal J. Ryan, Damian P. Drew, Chloe Douglas, Lex E. X. Leong, Max Moldovan, Miriam Lynn, Naomi Fink, Anastasia Sribnaia, Irmeli Penttila, Andrew J. McPhee, Carmel T. Collins, Maria Makrides, Robert A. Gibson, Geraint B. Rogers, David J. Lynn

**Affiliations:** a Precision Medicine Theme, South Australian Health and Medical Research Institute, Adelaide, South Australia, Australia; b College of Medicine and Public Health, Flinders University, Bedford Park, South Australia, Australia; c SAHMRI Women and Kids, South Australian Health and Medical Research Institute, Adelaide, South Australia, Australia; d Adelaide Medical School, The University of Adelaide, Adelaide, South Australia, Australia; e School of Agriculture, Food, and Wine, The University of Adelaide, Adelaide, South Australia, Australia; f Neonatal Medicine, Women’s and Children’s Hospital, North Adelaide, South Australia, Australia; Oregon State University

**Keywords:** BPD, RNA-Seq, VLBW, fecal organisms, microbiota, neonates

## Abstract

Bronchopulmonary dysplasia (BPD) is a common chronic lung condition in preterm infants that results in abnormal lung development and leads to considerable morbidity and mortality, making BPD one of the most common complications of preterm birth. We employed RNA sequencing and 16S rRNA gene sequencing to profile gene expression in blood and the composition of the fecal microbiota in infants born at <29 weeks gestational age and diagnosed with BPD in comparison to those of preterm infants that were not diagnosed with BPD. 16S rRNA gene sequencing, performed longitudinally on 255 fecal samples collected from 50 infants in the first months of life, identified significant differences in the relative levels of abundance of *Klebsiella, Salmonella, Escherichia/Shigella*, and *Bifidobacterium* in the BPD infants in a manner that was birth mode dependent. Transcriptome sequencing (RNA-Seq) analysis revealed that more than 400 genes were upregulated in infants with BPD. Genes upregulated in BPD infants were significantly enriched for functions related to red blood cell development and oxygen transport, while several immune-related pathways were downregulated. We also identified a gene expression signature consistent with an enrichment of immunosuppressive CD71^+^ early erythroid cells in infants with BPD. Intriguingly, genes that were correlated in their expression with the relative abundances of specific taxa in the microbiota were significantly enriched for roles in the immune system, suggesting that changes in the microbiota might influence immune gene expression systemically.

**IMPORTANCE** Bronchopulmonary dysplasia (BPD) is a serious inflammatory condition of the lung and is the most common complication associated with preterm birth. A large body of evidence now suggests that the gut microbiota can influence immunity and inflammation systemically; however, the role of the gut microbiota in BPD has not been evaluated to date. Here, we report that there are significant differences in the gut microbiota of infants born at <29 weeks gestation and subsequently diagnosed with BPD, which are particularly pronounced when infants are stratified by birth mode. We also show that erythroid and immune gene expression levels are significantly altered in BPD infants. Interestingly, we identified an association between the composition of the microbiota and immune gene expression in blood in early life. Together, these findings suggest that the composition of the microbiota may influence the risk of developing BPD and, more generally, may shape systemic immune gene expression.

## Abstract (precis)

Bronchopulmonary dysplasia (BPD) is a serious inflammatory condition of the lung and is the most common complication associated with preterm birth. A large body of evidence now suggests that the gut microbiota can influence immunity and inflammation systemically; however, the role of the gut microbiota in BPD has not been evaluated to date. Here, we report that there are significant differences in the gut microbiota of infants born at <29 weeks gestation and subsequently diagnosed with BPD, which are particularly pronounced when infants are stratified by birth mode. We also show that erythroid and immune gene expression levels are significantly altered in BPD infants. Interestingly, we identified an association between the composition of the microbiota and immune gene expression in blood in early life. Together, these findings suggest that the composition of the microbiota may influence the risk of developing BPD and, more generally, may shape systemic immune gene expression.

## INTRODUCTION

Bronchopulmonary dysplasia (BPD) is a chronic lung condition affecting approximately two-thirds of extremely preterm (born at <28 weeks gestational age) infants (1–3). BPD results in abnormal lung development and considerable morbidity, with complications continuing into adulthood (1). BPD was first described in 1967, but its clinical definition has since undergone several refinements. BPD as described now reflects an interruption to lung development (1, 4). BPD diagnosis and assessment of severity are based on the requirement for supplemental oxygen at 36 weeks postmenstrual age (gestational age plus postnatal age) (3).

The pathogenesis of BPD is recognized to be multifactorial, with potential impacts of both pre- and postnatal conditions on its development and severity (3, 5). Currently, gestational age and birth weight remain the best described risk factors for BPD (3, 6), and therefore, there is significant clinical interest in identifying other factors that can more accurately predict which infants are at risk of developing BPD. Several studies have identified a large contribution of genetic factors to BPD susceptibility, with 53 to 82% of the variance explained by genetics in twin cohort studies in North America (7, 8). Genome-wide association studies (GWAS) of BPD, however, have produced inconclusive results, with one study finding an association with the SPOCK2 gene and another with the C-reactive protein (CRP) gene but others finding no significant associations (9–12). The association of rare variants with BPD has been evaluated through multiple exome sequencing studies, which have suggested a role for kinase A- and mitogen-activated protein (MAP) kinase-related pathways in BPD (13–15). To date, microarray-based gene expression studies of BPD have been performed on both blood and lung tissue samples collected from infants and from animal models and have identified several thousand genes as potentially being differentially expressed in BPD (16–19). These studies reported that pathways involved in the inflammatory response are downregulated in BPD infants, while pathways related to the cell cycle are upregulated (18). These signatures were, however, dependent on the timing of sample collection for gene expression analysis.

The gut microbiota in very low birth-weight (VLBW; born weighing <1,500 g) preterm infants has been shown to follow a markedly different pattern of colonization than that of healthy term-born infants (20–22), leading us to hypothesize that the gut microbiota could influence BPD susceptibility and/or severity. Consistent with this hypothesis, prolonged antibiotic use in VLBW preterm infants is associated with an increased risk of developing BPD (23, 24). Recent studies have identified differences in the lung microbiota in infants who develop BPD (25); however, to date, only one small (*n* = 13) PCR-based study has investigated whether the composition of the gut microbiota was altered in infants with BPD (26). Increasing evidence from preclinical and clinical studies strongly suggests that the gut microbiota plays a key role in healthy immune development, particularly in early life (27, 28). Importantly, several studies have demonstrated the potential of the gut microbiota to influence immune responses in the lung (28, 29), suggesting that the gut microbiota could influence the severity of BPD by modulating inflammatory responses systemically and in the lung.

To investigate whether changes in the gut microbiota are associated with BPD, we used 16S rRNA gene sequencing to perform longitudinal profiling of the microbiota in >250 fecal samples collected from a cohort of 50 infants born at <29 weeks gestation. We identified significant differences in the relative abundances (RAs) of several taxa in the fecal microbiota of vaginally born infants that were subsequently diagnosed with BPD. Interestingly, these differences were not evident in infants born by cesarean section. Blood samples were also collected from a subset of the infants at baseline and at approximately the time that BPD diagnosis was made. Gene expression in venous blood collected from BPD infants was not significantly different at baseline compared to the gene expression in venous blood from non-BPD infants, but >400 genes were significantly differentially expressed at the time of BPD diagnosis. We identified a gene expression signature in BPD infants that suggests an enrichment of immunosuppressive CD71^+^ early erythroid cells. Finally, we also uncovered a potential association between the composition of the microbiota and peripheral blood immune gene expression.

## RESULTS

**FIG 1:**
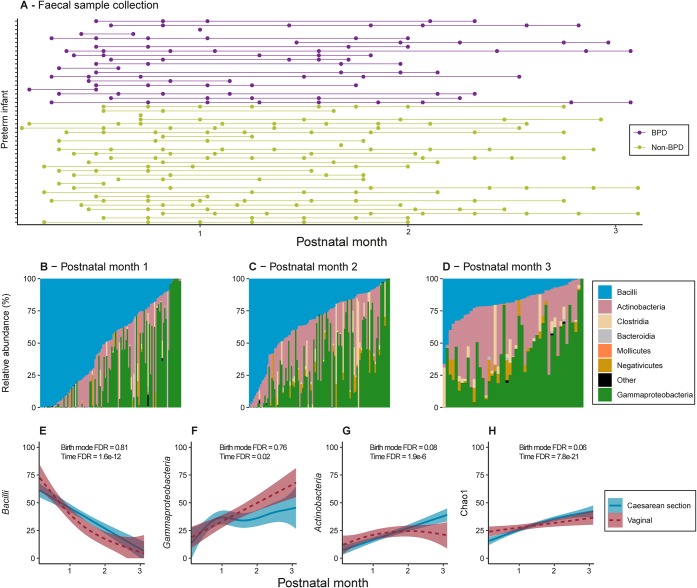
16S rRNA gene sequencing was used to longitudinally profile the composition of the fecal microbiota in 50 preterm infants subsequently diagnosed with or without BPD. (A) Filled circles represent time points at which fecal samples were collected from each infant (mean of 5 per infant, total of 255). (B to D) The relative abundances of the major classes of bacteria identified in the preterm infants in this study. (E to G) The mean relative abundances (line graph) of the top three most abundant bacterial classes over time in preterm infants that were born vaginally or by cesarean section. Shaded areas represent the 95% confidence intervals. (H) Chao1 alpha diversity. A generalized linear mixed-effects model was used to assess changes in relative abundance or alpha diversity over time (Time FDR) or by birth mode (Birth mode FDR).

Two hundred fifty-five fecal samples (mean of 5 per infant) were collected from 50 preterm infants (mean gestational age, 26.4 ± 1.7 weeks [mean ± standard deviation]; mean birth weight, 869.9 ± 240.1 g), recruited from the Women’s and Children’s Hospital, Adelaide, Australia. Samples were collected longitudinally from recruitment (shortly after birth) to discharge home or 40 weeks postmenstrual age, whichever occurred first (Fig. 1A). These infants were recruited as a subset of infants enrolled in the larger, multisite n-3 Fatty Acids for Improvement in Respiratory Outcomes (N3RO) clinical trial, which investigated the impact of enteral docosahexaenoic acid (DHA) supplementation on the risk of developing BPD (30). BPD was defined as the requirement for supplemental oxygen and/or respiratory support at 36 weeks’ postmenstrual age or discharge home, whichever occurred first, using modified criteria (see Materials and Methods) from Walsh et al. (31). A subset of infants in this study also had venous blood collected for gene expression profiling via RNA-Seq at recruitment/baseline (*n* = 10; mean day of life, day 4.6) and again at the time of BPD diagnosis (*n* = 21; mean day of life, day 64.4) (Fig. 1A; see Table S1 in the supplemental material).

10.1128/mSystems.00484-19.7 TABLE S1 Sample and subject metadata. Download Table S1, XLSX file, 0.02 MB. Copyright © 2019 Ryan et al. 2019 Ryan et al. This content is distributed under the terms of the Creative Commons Attribution 4.0 International license.

Of the 50 infants recruited, hereinafter referred to as BPD/non-BPD infants, 20 were subsequently diagnosed with BPD at 36 weeks postmenstrual age. One infant died prior to 36 weeks postmenstrual age and did not receive a BPD diagnosis. Another infant could not be diagnosed with/without BPD as the physiological challenge was not performed (although the infant was receiving respiratory support at 36 weeks postmenstrual age). Fecal microbiota data from these two infants were excluded when testing for associations with BPD diagnosis. Ten of the 20 BPD infants and 15/28 non-BPD infants received DHA supplementation. As is common for this population, all but two received antibiotics over the course of the study, both of whom were non-BPD cesarean section-born infants. Similarly, all but two infants (one BPD and one non-BPD, both cesarean section born) received the probiotic Infloran (Berna, Switzerland), a mixture of *Bifidobacterium* and *Lactobacillus* species. Probiotics are commonly administered to preterm infants in Australia to reduce necrotizing enterocolitis (32, 33). There was no significant difference in the number of days of probiotic supplementation between the BPD diagnosis groups (data not shown). Lower gestational age is a well-documented risk factor for BPD. While all infants in this cohort were born very premature, BPD infants were on average 1.2 weeks more preterm than non-BPD infants (mean of 25.6 versus 26.8 weeks, *P* = 0.017). Further cohort demographics are shown in Table 1.

**TABLE 1:** Infant clinical data according to BPD diagnosis

Characteristic	BPD (*n* = 20)	Non-BPD (*n* = 28)	Unadjusted *P* value
Mean gestational age ± SD (wks)	25.7 ± 1.8	26.8 ± 1.4	0.018^*a*^
Mean birth wt ± SD (g)	746.8 ± 187.4	967.9 ± 233.5	0.00095^*a*^
Female (%)	45	46.4	1^*b*^
Vaginally born (%)	40	28.5	0.537^*b*^
Sepsis (% yes)	35	17.9	0.19^*b*^
Necrotizing enterocolitis (% yes)	10	3.6	0.57^*b*^
Mean no. of fecal samples per subject ± SD	5.0 ± 2.1	5.9 ± 2.4	0.29^*a*^
Postnatal steroid treatment (% yes)	60	10.7	0.0004^*b*^

a *P* value was generated by Wilcoxon test for continuous variables.

b *P* value was generated by Fisher’s exact test for categorical variables.

### Composition of the fecal microbiota in VLBW preterm infants.

The fecal microbiota of the preterm infants in this study were assessed by 16S rRNA gene sequencing. Samples were sequenced to an average of 31,464 (2 × 300 bp) reads on an Illumina MiSeq (interquartile range [IQR], 11,779). The DADA2 algorithm (34), as implemented in QIIME2 (35), was used to denoise sequences, resulting in a total of 676 unique ribosomal sequence variants that were detected in at least 1 sample (hereinafter referred to as exact sequence variants [ESVs]). Six samples that had <1,000 denoised reads were excluded from further analysis. Multiple fecal samples were collected per infant, allowing a longitudinal assessment of changes in the composition of the microbiota during the first months of life (Fig. 1B to D; Fig. S1).

10.1128/mSystems.00484-19.1 FIG S1 The smoothed mean relative abundances of bacterial phyla, classes, orders, families, and genera in BPD (purple) and non-BPD (green) infants. Solid lines represent the mean relative abundances of phyla in cesarean section-born infants; dashed lines represent vaginally born infants. Shaded areas represent the 95% confidence intervals. Only taxa with mean relative abundances greater than 1% are shown. Download FIG S1, PDF file, 0.4 MB. Copyright © 2019 Ryan et al. 2019 Ryan et al. This content is distributed under the terms of the Creative Commons Attribution 4.0 International license.

In the first month of life, three bacterial classes dominated the fecal microbiota: *Bacilli* (mean relative abundance [RA], 38.14%; IQR, 43.94%), *Gammaproteobacteria* (mean RA, 35.19%; IQR, 57.42%), and *Actinobacteria* (mean RA, 20.23%; IQR, 32.95%). After the first month of life, the *Bacilli* RA decreased sharply, while the RAs of both the *Gammaproteobacteria* and *Actinobacteria* increased (Fig. 1E to G). *Actinobacteria* were observed to be highly abundant, which may be a result of the Infloran probiotic that was administered to all but 2 of the infants. The increased RA of *Actinobacteria* observed in this study was consistent with previous reports demonstrating significant increases in the RAs of *Bifidobacterium* in the microbiota of infants receiving *Bifidobacterium*-based probiotics (36). *Clostridia* have previously been reported to be highly abundant in the VLBW preterm infant fecal microbiota, at levels comparable to the levels of *Gammaproteobacteria* and *Bacilli* (20, 37). In our study, only a small subset of infants had a high RA of *Clostridia*, while in most infants, they were either not detected or were present at a low RA (mean RA, 3.55%; IQR, 1.35%) (Fig. 1B to D; Fig. S1). Alpha diversity (Chao1 index) increased with age (Fig. 1H), as has been previously reported (37).

Due to the limitations of species-level taxonomic classification with the V4 region of the 16S rRNA gene (38), we examined subgenus-level variation by defining coabundant groups (CAGs) of ESVs based on their Spearman correlations (39). Correlation analysis, limited to ESVs present in at least 20 samples, indicated that ESV-level abundance could largely be attributed to 16 highly correlated CAGs of ESVs (Fig. S2A). While certain genera were spread over multiple CAGs (*Bifidobacterium*, *Streptococcus*, and *Lactobacillus*), the majority of CAGs were made up of ESVs which could be classified as either a single bacterial genus or a family. This is in contrast to the complex coabundance relationships commonly observed in the adult microbiota (40). The intra-CAG taxonomic homogeneity and high intra-CAG correlation observed may indicate that CAGs represent individual species (or groups of species not discernible with the taxonomic resolution of 16S rRNA gene sequencing). Sequences classified as part of the family *Enterobacteriaceae* were also spread across multiple CAGs that showed little to no correlation with each other (Fig. S2A). Examination of CAG RAs over time indicated that CAGs largely capture the same variation as shown at the genus level of classification (Fig. S2B), with the exception of those taxa mentioned above.

10.1128/mSystems.00484-19.2 FIG S2 Coabundances of 16S rRNA exact sequence variants (ESVs). (A) Heatmap of the Spearman correlation between ESVs that were present in at least 20 samples. The heatmap was generated using the made4 package in R. (B) The smoothed mean relative abundances of bacterial CAGs in BPD (purple) and non-BPD (green) infants. Solid lines represent the mean relative abundances of bacterial CAGs in cesarean section-born infants; dashed lines represent vaginally born infants. Shaded areas represent the 95% confidence intervals. Only bacterial CAGs with mean relative abundances greater than 0.5% are shown. Download FIG S2, PDF file, 0.4 MB. Copyright © 2019 Ryan et al. 2019 Ryan et al. This content is distributed under the terms of the Creative Commons Attribution 4.0 International license.

### Differences in the compositions of the fecal microbiota in preterm infants diagnosed with BPD.

**FIG 2:**
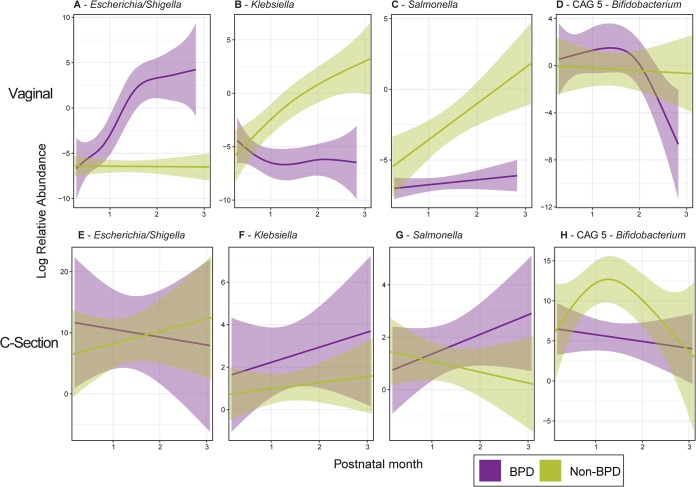
Bacterial genera and CAGs that were identified as being significantly associated (FDR ≤ 0.01) with BPD diagnosis using a generalized linear mixed-effects model. The model was implemented using the lme4 package in R and incorporated day of life, birth mode, BPD diagnosis, DHA treatment, and subject (as a random effect). Three genera were found to be significantly associated with BPD, *Escherichia/Shigella* (FDR = 5.27e−08), *Klebsiella* (FDR = 0.0059), and *Salmonella* (FDR = 0.014), as well as a single *Bifidobacterium* CAG (FDR = 0.0039). (A to D) Log relative abundances of genera associated with BPD in fecal samples collected from BPD and non-BPD vaginally born infants. (E to H) Log relative abundances of genera associated with BPD in fecal samples collected from BPD and non-BPD cesarean section-born infants. Smoothed mean values (line graph) and 95% confidence intervals (shaded areas) are shown.

To identify taxa in the fecal microbiota that were significantly altered in BPD infants over time, we implemented a generalized linear mixed-effects model (GLMM) using the lme4 R package (41). The longitudinal RAs of taxa at the class and genus levels, as well as CAG RAs, were analyzed for an association with BPD diagnosis while controlling for day of life, birth mode, DHA treatment, and subject. Three genera (*Escherichia/Shigella*, *Klebsiella*, and *Salmonella*) from the *Enterobacteriaceae* (class *Gammaproteobacteria*) were significantly associated (false discovery rate [FDR] ≤ 0.01) with BPD diagnosis (Fig. 2A, C, and E to G). Interestingly, these associations were only evident in vaginally born BPD infants. *Escherichia/Shigella* were significantly increased in vaginally born BPD infants but were not significantly altered in cesarean section-born infants, while *Klebsiella* and *Salmonella* were significantly less abundant in vaginally born (but not cesarean section-born) BPD infants. Additionally, the RA of one of the two *Bifidobacterium* CAGs (CAG 5) was significantly associated with BPD diagnosis in cesarean section-born infants (FDR = 0.0028) (Fig. 2D and H; Table S2). The GLMM approach did not identify any significant association between BPD diagnosis and any taxon at the higher taxonomic levels or with alpha diversity (Table S2).

10.1128/mSystems.00484-19.8 TABLE S2 FDR-corrected *P* values from generalized linear mixed model tests of relative abundance. Subject was modelled as a random effect, while BPD and DHA treatment were included as fixed effects. Download Table S2, XLSX file, 0.01 MB. Copyright © 2019 Ryan et al. 2019 Ryan et al. This content is distributed under the terms of the Creative Commons Attribution 4.0 International license.

As antibiotics are among the most frequently prescribed medications in neonatal intensive care units (42), we sought to establish whether a relationship existed between antibiotic treatment and the relative abundances of BPD-associated taxa. All but two infants in this cohort received antibiotics; however, vaginally born infants in this cohort had a significantly higher number of days of antibiotic exposure than those born by cesarean section, both in the first week of life and over the entire study period (Fig. S3A and C). There were no significant differences in numbers of days of antibiotic exposure by BPD diagnosis, either in the first week of life or over the entire study period (Fig. S3B and D). Importantly, there was no significant difference in numbers of days of antibiotic exposure in vaginally born BPD infants compared to the exposure of vaginally born non-BPD infants, suggesting that antibiotic exposure cannot explain the association between the composition of the fecal microbiota and BPD diagnosis observed in these infants. Furthermore, we examined the correlation between the numbers of days of antibiotic exposure and the RAs over time (summarized by the area under the curve [AUC] for each taxon) of each genus and CAG that we found to be associated with BPD in the vaginally born infants. No significant correlation between the number of days of antibiotic exposure and the RA of any of the taxa associated with BPD was found (Fig. S3E to H).

10.1128/mSystems.00484-19.3 FIG S3 Days of antibiotic exposure. (A) Days of antibiotic exposure in the first week of life in vaginally born versus cesarean section-born preterm infants. (B) Days of antibiotic exposure in the first week of life in vaginally born versus cesarean section-born BPD and non-BPD preterm infants. (C) Days of antibiotic exposure over the entire study period in vaginally born versus cesarean section-born preterm infants. (D) Days of antibiotic exposure over the entire study period in vaginally born versus cesarean section-born BPD and non-BPD preterm infants. A Wilcoxon test was used to assess statistical significance. The lengths of the boxplot whiskers represent 1.5 times the interquartile range. (E to H) Pearson correlation analysis of taxa found to be associated with BPD diagnosis and days of antibiotic exposure. Taxon relative abundances over time were summarized to a single value per subject as area under curve (AUC). Smoothed mean values (line graphs) and 95% confidence intervals (shaded areas) are shown. Download FIG S3, PDF file, 0.1 MB. Copyright © 2019 Ryan et al. 2019 Ryan et al. This content is distributed under the terms of the Creative Commons Attribution 4.0 International license.

### Impact of DHA supplementation on the fecal microbiota.

Infants in the N3RO study were randomized to receive a daily enteral soy-based placebo or supplementation with 60 mg of DHA per kg of body weight within 3 days of commencing enteral feeding, finishing at 36 weeks postmenstrual age (mean of 60 days of DHA supplementation). In the larger N3RO trial, supplementation with DHA did not decrease and may have increased the risk of developing BPD (30). Interestingly, using the GLMM approach described above, we found a significant association between DHA treatment and the RA of *Escherichia/Shigella* that was independent of BPD diagnosis (FDR = 4.32e−8) (Fig. S4). These data suggest that the RA of *Escherichia/Shigella* is associated with both BPD diagnosis and DHA treatment. We detected no significant association between DHA treatment and alpha or beta diversity (Fig. S4C and D).

10.1128/mSystems.00484-19.4 FIG S4 The relative abundances of *Escherichia/Shigella* were significantly associated (FDR < 0.01) with DHA supplementation. (A) Vaginally born infants. (B) Cesarean section-born infants. Smoothed mean relative abundances (line graph) and 95% confidence intervals (shaded areas) are shown. (C) Principal component analysis of weighted UniFrac distances. Shaded areas represent the 95% confidence regions. (D) Alpha diversity (Chao1 index). The Wilcoxon test was used to assess statistical significance. The lengths of the boxplot whiskers represent 1.5 times the interquartile range. Download FIG S4, PDF file, 0.1 MB. Copyright © 2019 Ryan et al. 2019 Ryan et al. This content is distributed under the terms of the Creative Commons Attribution 4.0 International license.

### Differential gene expression in preterm infants diagnosed with BPD.

**FIG 3:**
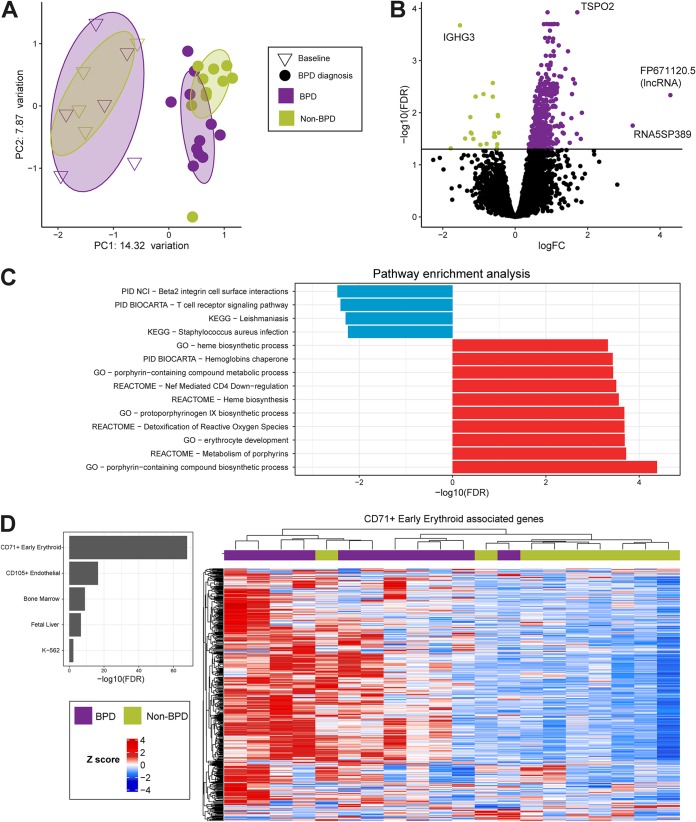
RNA-Seq was used to profile gene expression in peripheral blood samples collected from BPD and non-BPD infants at recruitment (baseline) and at the time of BPD diagnosis. (A) Multidimensional scaling (MDS) analysis of RNA-Seq data in BPD and non-BPD infants at baseline (mean day of life, day 4.6) and at the time of BPD diagnosis (mean day of life, day 64.4). PC1 and -2, principal coordinates 1 and 2. (B) Volcano plot showing genes that were differentially expressed at the time of BPD diagnosis in BPD versus non-BPD infants. Horizontal line corresponds to an FDR value of 0.05. Positive log fold change (logFC) values correspond to genes with increased expression in BPD relative to non-BPD infants. (C) Top pathways and GO terms enriched among differentially expressed genes. Red bars represent pathways/terms upregulated in BPD. Blue bars represent downregulation. (D) Right, heatmap showing the expression levels of CD71^+^ early erythroid cell-associated genes at time of BPD diagnosis; bottom left, heat map key; top left, CTen enrichment analysis results.

Gene expression in peripheral venous blood samples collected from a subset of infants at baseline (*n* = 10; mean day of life, day 4.6) and again at BPD diagnosis (*n* = 21; mean day of life, day 64.4), was profiled using RNA-Seq. More than 1.2 billion reads were sequenced (mean, 56 million single-end, 100-bp reads per sample; IQR, 11.3 million reads). Reads were aligned to the human genome (GRCh38) using HISAT2, with a mean alignment rate of 95.6% (Table S1). Infant gene expression profiles clustered primarily by age at time of blood collection, indicating that large-scale differences in blood gene expression occur between the first week of life and time of BPD diagnosis (Fig. 3A). One non-BPD infant was a notable outlier in gene expression compared to the other non-BPD infants (Fig. 3A). While this infant did not meet either the physiological or the clinical definition of BPD used in the N3RO clinical trial (30), this infant received 61 days of supplemental oxygen, which put this infant in the 92nd percentile of supplemental oxygen among all non-BPD infants in the study. We excluded this infant from further analysis to avoid confounding results. More than 2,000 genes were identified as differentially expressed at 36 weeks postmenstrual age compared to baseline (Table S3). No genes differentially expressed between BPD and non-BPD infants were identified in samples collected at baseline (FDR < 0.05). In contrast, 431 genes were identified as being significantly differentially expressed in BPD infants compared to their expression levels in non-BPD infants at time of diagnosis (FDR < 0.05) (Fig. 3B; Table S3). Pathway and Gene Ontology (GO) analyses revealed that BPD infants had increased expression of genes involved in red blood cell development and oxygen transport, whereas immune-related pathways were downregulated (Fig. 3C; Table S3). Postnatal steroids may be administered to preterm infants as a treatment for chronic lung disease (43), and as expected, BPD infants received significantly more steroids than non-BPD infants (Table 1). While increased steroid administration could contribute to the observed downregulation of immune-related genes in BPD infants (44), we observed a similar pattern of expression in BPD infants regardless of postnatal steroid treatment (Fig. S5A). Changes in whole blood gene expression could reflect changes in the frequencies of immune or erythroid cell populations. The small volume of the blood samples collected from the VLBW preterm infants precluded analysis by flow cytometry. To computationally assess whether specific cell populations were enriched among genes upregulated in BPD infants, cell type enrichment analysis was performed using the CTen (Cell Type ENrichment) platform (45). This analysis revealed that genes upregulated in BPD were significantly enriched for genes expressed in CD71^+^ early erythroid cells (Fig. 3D; Fig. S5B). Transcription factor binding site analysis identified a single motif enriched among the promoters of genes upregulated in BPD (FDR < 0.05). The motif GATA:SCL was found in 4.53% of those upregulated genes but only 0.89% of background sequences. GATA motifs have been found to regulate hematopoietic development (46), which is consistent with the upregulation of genes involved in red blood cell development.

10.1128/mSystems.00484-19.5 FIG S5 Heatmaps of BPD-associated genes and infant metadata (A) Heatmap showing the expression levels at the time of BPD diagnosis of 431 genes that were identified as being significantly differentially expressed (FDR < 0.05) in BPD infants. (B) Heatmap showing the expression levels of CD71^+^ early erythroid-associated genes. Download FIG S5, PDF file, 0.3 MB. Copyright © 2019 Ryan et al. 2019 Ryan et al. This content is distributed under the terms of the Creative Commons Attribution 4.0 International license.

10.1128/mSystems.00484-19.9 TABLE S3 Differential gene expression analysis results from EdgeR and InnateDB pathways and Gene Ontology terms enriched among genes that were differentially expressed in BPD infants. Download Table S3, XLSX file, 0.3 MB. Copyright © 2019 Ryan et al. 2019 Ryan et al. This content is distributed under the terms of the Creative Commons Attribution 4.0 International license.

### Specific taxa in the preterm infant gut microbiota are correlated with gene expression in blood.

Given the increasingly well-recognized influence of the gut microbiota on immune responses in the periphery (29), particularly in early life (28), we speculated that the composition of the gut microbiota in preterm infants might be associated with changes in (immune) gene expression in blood at the time of BPD diagnosis. A partial Spearman correlation analysis, which controlled for birth mode, sex, and gestational age, was used to assess the association between CAG relative abundance and normalized blood gene expression. This analysis was not limited to only immune gene expression but considered all genes that were at least modestly expressed in blood (count per million [cpm] of >10 in 15 samples). The approach used was similar to that used in a publication from the Human Microbiome Project (47). Due to the relatively small sample size, all infants (i.e., both BPD and non-BPD) with 16S rRNA gene sequencing and RNA sequencing data were included in the analysis. Our analysis was not sufficiently powered to identify statistically significant individual gene-CAG correlations after correction for multiple testing. Instead, we assessed whether genes identified as being correlated with CAG relative abundance at a *P* value of <0.05 were enriched for specific biological pathways or processes. This approach is analogous to methods that have been successfully adopted to detect statistical signatures in genome-wide association (GWA) data when individual associations do not reach genome-wide significance (48–51). Additionally, the statistical significance of enriched terms was further assessed using a permutation-based approach, whereby we randomized the gene labels and repeated the enrichment analysis 1,000 times. Any pathways/GO terms found to be significantly enriched (FDR < 0.01) in any permutation with randomized gene labels were not considered to be significantly associated with CAG relative abundance.

**FIG 4:**
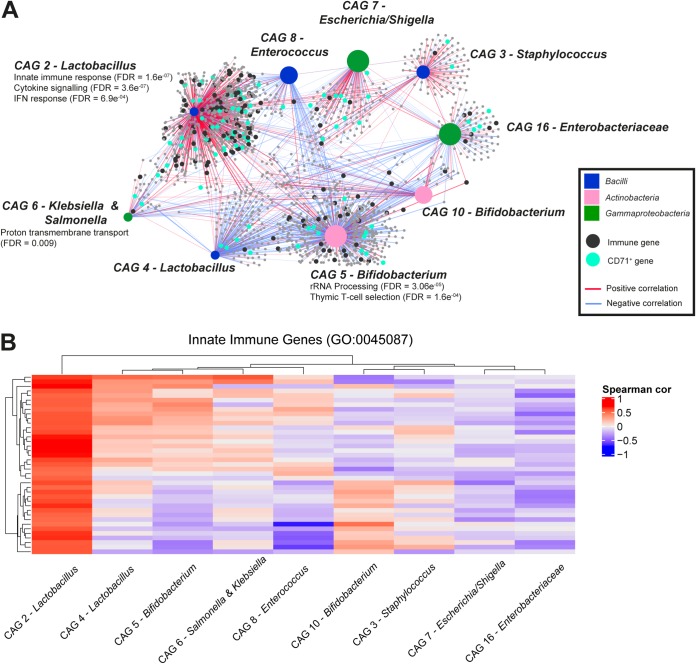
Correlation analysis between microbiota relative abundances and gene expression levels at time of BPD diagnosis. (A) Network showing genes expressed in blood at 36 weeks postmenstrual age that were correlated (*P* < 0.05) in their expression with the relative abundance of at least one fecal microbiota coabundant group (CAG) in postnatal month 2 (larger colored nodes). Gene nodes are colored according to whether they are immune associated (i.e., in GO terms GO:0002376, GO:0045087, or GO:0006955) or associated with CD71^+^ early erythroid cells. The size of each CAG node is proportional to its relative abundance. Pathways and GO terms that were significantly enriched (FDR < 0.01) among genes correlated with the relative abundances of specific CAG nodes are shown. See Table S4 in the supplemental material for further details. IFN, interferon. (B) Heatmap of the Spearman correlation coefficients between innate immune genes (GO:0045087) and CAG relative abundances in postnatal month 2. Only genes that were correlated (*P* < 0.05) with at least one CAG are shown.

Given the multiple samples collected longitudinally per infant, the highly variable nature of the composition of the microbiota in early life, and reports of time-dependent microbiota-immune interactions (52), we performed these analyses independently for each month of life (selecting the last sample collected per month for each infant). First, we investigated which pathways or GO terms were statistically enriched among genes correlated with the RA of at least one CAG (Table S4). We found that genes correlated with CAG RAs were significantly enriched for multiple immune response pathways and GO terms (Table S4). Next, we assessed genes correlated with the relative abundances of specific CAGs and found that genes correlated with 7 of the 16 CAGs were enriched for a range of immune processes (Fig. 4; Fig. S6A and B; Table S4). For example, genes negatively correlated with the RA of *Bifidobacterium* in the first month of life were enriched for inflammatory response genes (FDR = 5.1e−09) (Table S4), suggesting that a higher relative abundance of *Bifidobacterium* might be associated with lower inflammatory gene expression in blood. Consistent with these data, the potential anti-inflammatory effects of *Bifidobacterium* have been demonstrated in a number of studies (53).

10.1128/mSystems.00484-19.6 FIG S6 Correlation analysis between microbiota relative abundances and gene expression levels at time of BPD diagnosis. (A) Network showing genes expressed in blood at 36 weeks postmenstrual age that were correlated (*P* < 0.05) in their expression with the relative abundance of at least one fecal microbiota coabundant group (CAG) in postnatal month 1 (larger colored nodes). (B) Network showing genes expressed in blood at 36 weeks postmenstrual age that were correlated (*P* < 0.05) in their expression with the relative abundance of at least one fecal microbiota CAG in postnatal month 3 (larger colored nodes). Immune genes (i.e., genes annotated with GO terms GO:0002376, GO:0045087, or GO:0006955) and genes associated with CD71^+^ early erythroid cells are highlighted. The size of each CAG node is proportional to its relative abundance. Pathways and GO terms that were significantly enriched (FDR < 0.01) among genes correlated with the relative abundances of specific CAG nodes are shown. See Table S4 for further details. (C) The expression levels of type I interferon-inducible genes in blood at 36 weeks postmenstrual age were correlated (*P* < 0.05) with the relative abundance of a *Lactobacillus* CAG in postnatal month 2. Shaded areas indicate 95% confidence intervals. Download FIG S6, PDF file, 2.7 MB. Copyright © 2019 Ryan et al. 2019 Ryan et al. This content is distributed under the terms of the Creative Commons Attribution 4.0 International license.

10.1128/mSystems.00484-19.10 TABLE S4 GO terms and pathways found to be significantly enriched among gene sets identified as correlated with the relative abundances of CAGs in the fecal microbiota. Download Table S4, XLSX file, 0.02 MB. Copyright © 2019 Ryan et al. 2019 Ryan et al. This content is distributed under the terms of the Creative Commons Attribution 4.0 International license.

Genes correlated with the RA of *Lactobacillus* in the second month of life of life also showed a strong enrichment for immune-related genes, including genes involved in interferon signaling (Fig. 4A) and the innate immune response (Fig. 4B). Different *Lactobacillus* strains have been shown to induce type I and II interferons in multiple previous human and mouse studies (54–57). The strong correlation between type I interferon-inducible gene expression and the RA of *Lactobacillus* was also evident at the per-gene level (Fig. S6C). We also identified an association between the RA of *Staphylococcus* in the third month of life and other immune-related processes implicated in the pathogenesis of BPD, such as the inflammatory response and MAP kinase activation (Fig. S6B; Table S4). Finally, we evaluated whether any of the correlated gene sets were also enriched for CD71^+^ early erythroid cell-associated genes. This cell type has been shown to regulate inflammation induced by the microbiota in neonates (58). Genes correlated with the RAs of 5 of the CAGs were found to be significantly enriched for CD71^+^-associated genes. Multiple members of the *Bacilli* showed particularly strong enrichments for CD71^+^-associated genes across multiple months (Table S4), which is consistent with previous reports that members of this bacterial class can induce the expression of CD71^+^-associated genes (59).

While this analysis does not prove a causative link between the composition of the microbiota and gene expression in blood, our approach has identified a relatively small set of host-microbe associations, some of which are supported by the literature, that may help in understanding the role the microbiota plays in shaping the immune system in preterm infants. Further work is now needed to validate these associations in animal models and better-powered clinical studies.

## DISCUSSION

Bronchopulmonary dysplasia (BPD) is a chronic inflammatory condition of the lung that is one of the most common complications associated with early preterm birth. A large body of evidence now suggests that the gut microbiota can influence immunity and inflammation systemically, including in the lung (28, 29). The composition of the gut microbiota in early life has also been associated with the risk of necrotizing enterocolitis and sepsis (60–62). However, whether the gut microbiota influences susceptibility to BPD is currently unknown. To investigate whether changes in the gut microbiota are associated with BPD, we used 16S rRNA gene sequencing to longitudinally profile the composition of the microbiota in >250 fecal samples collected from a cohort of 50 preterm infants born at <29 weeks gestation. Adjusting for several potentially confounding factors, we identified three genera (*Escherichia/Shigella*, *Klebsiella*, and *Salmonella*) from the *Enterobacteriaceae* (class *Gammaproteobacteria*) that were significantly associated with BPD diagnosis, but interestingly, these differences were only evident in vaginally born BPD infants. Members of the *Gammaproteobacteria* have previously been identified to be associated with vaginal birth in VLBW infants (37). The relative abundance of one of the *Bifidobacterium* CAGs was also significantly associated with BPD diagnosis. All four of the genera that showed significant association with BPD diagnosis have previously been identified as colonizers of the VLBW infant gut microbiota (20–22, 37), with Escherichia coli in particular being implicated in both necrotizing enterocolitis (62) and late-onset sepsis (63) in preterm infants. Differences in antibiotic exposures, which were higher in vaginally born infants, could also potentially explain why different associations were detected in vaginally and cesarean section-born infants. Importantly, however, there was no significant difference in antibiotic or probiotic exposure between vaginally born BPD and non-BPD infants, indicating that these factors cannot fully explain associations between taxa in the fecal microbiota and BPD diagnosis. Previous studies of the VLBW infant gut microbiota have primarily focused on investigating changes in the gut microbiota at the phylum or class level (20, 21, 37), but these data show that important intrafamily and intragenus variation may be present, suggesting that methods allowing higher taxonomic resolution should be implemented where possible.

Aside from the differences between BPD- and non-BPD-diagnosed infants, the preterm infants in our study displayed a pattern of colonization over time broadly similar to that described previously, with *Gammaproteobacteria* increasing with age as *Bacilli* sharply decreased (20–22, 37). Interestingly, *Clostridia* were absent from the majority of infants in our cohort, whereas *Clostridia* have been reported to be a major constituent of the VLBW preterm infant fecal microbiota in other studies (20–22, 37). We also observed much higher levels of *Actinobacteria* than have previously been reported (20–22, 37). These differences in the relative abundances of *Actinobacteria* and *Clostridia* may in part be due to the administration of the probiotic Infloran, a mixture of Bifidobacterium bifidum, Bifidobacterium infantis, and Lactobacillus acidophilus (36). Probiotic supplementation has routinely been given to very preterm infants in Sweden, Japan, and other nations for over a decade (33) and, as of 2015, has been widely adopted in Australia for this population (33). Importantly, Infloran was administered to almost all infants in our study and, therefore, does not confound comparisons of the different infant groups.

In addition to profiling the composition of the microbiota, we also profiled gene expression in peripheral blood samples collected from a subset of infants at baseline and again at the time of BPD diagnosis. Gene expression profiles differed significantly between time points, in agreement with previous reports of changes in cell populations in neonatal blood during this developmental period (64). While there was no significant difference in gene expression between BPD and non-BPD infants at baseline, more than 400 genes were identified as differentially expressed at the time of BPD diagnosis. Using microarray analysis, Pietrzyk et al. reported that there were between 324 and 3,498 genes differentially expressed in BPD infant blood samples, depending on day of life (18). We identified considerably fewer alterations in gene expression; however, this may be in part due to different technologies used to profile gene expression (microarray versus RNA-Seq) or differences in the time points. Pathway overrepresentation analysis found a significant enrichment for processes associated with oxygen transport and red blood cell development among genes upregulated in BPD infants in our study. Consistent with these results, we also found an enrichment of a GATA motif, which has been associated with hematopoietic development, in the promoters of genes upregulated in BPD (46). Cell type enrichment analysis also revealed enrichment for CD71^+^ early erythroid cells among genes that were upregulated in BPD infants. CD71^+^ cells have been shown to prevent excessive inflammation induced by commensal microbes colonizing following birth (58). Future studies should assess changes in circulating cell populations in BPD infants using flow cytometry. Intriguingly, we also uncovered associations between the relative abundances of certain taxa in the microbiota and host gene expression levels in blood, including an association between *Lactobacillus* and interferon signaling, which is supported by multiple human and mouse studies showing that a number of different *Lactobacillus* strains can regulate interferon gene expression (54–57). This analysis also pointed to a number of associations between the microbiota and several pathways relevant to BPD pathogenesis and the expression of CD71^+^-associated genes. These data suggest that changes in the gut microbiota might influence immune gene expression systemically; however, these associations need to be replicated in further studies or in animal models before a causative link can be supported.

Our study is not without its limitations. As the gut microbiota at this stage of life is volatile, we focused on a modest number of infants (*n* = 50) with many repeated samples to assess whether changes in the gut microbiota are associated with BPD diagnosis. The sample size limits our statistical power to detect weaker associations, and further studies with larger sample sizes are now warranted to confirm our findings. BPD was diagnosed based on the requirement for supplemental oxygen at 36 weeks postmenstrual age based on an assessment of oxygen saturation in infants meeting prespecified criteria (65). Due to our relatively limited sample size, we did not assess the relationship between the microbiota and BPD subtypes. Furthermore, all infants were recruited from the neonatal intensive care unit (NICU) in a single hospital, which may have been the source of some commonly detected microbes. Replication of our findings in larger multicenter studies is a key next step. Preterm infants are routinely exposed to medication other than antibiotics, including corticosteroids, which may impact the composition of the microbiota (66). Assessing what role, if any, current treatment regimens play in shaping the BPD gut microbiota will be a crucial, albeit challenging task. Finally, 16S rRNA sequencing has limited species- and strain-level resolution (67), multiple copies of the 16S rRNA gene per genome may inflate diversity estimates (68), and the choice of primer set may impact taxon detection. Future studies should investigate the use of shotgun metagenomics approaches to profile the composition of the microbiota.

## MATERIALS AND METHODS

### Study design and sample collection.

The infants in this study were recruited as part of the n-3 Fatty Acids for Improvement in Respiratory Outcomes (N3RO) trial (30). In N3RO, infants born before 29 weeks gestation who had commenced enteral feeding in the previous 3 days were eligible to participate. Infants were randomized to receive an enteral emulsion of DHA (60 mg/kg of body weight/day) or a control emulsion (soy) from randomization to 36 weeks postmenstrual age. The primary outcome in N3RO was BPD, defined as the requirement for supplemental oxygen and/or respiratory support at 36 weeks postmenstrual age or discharge home, whichever occurred first, using modified criteria from Walsh et al. (31) involving a physiological challenge. For a complete description of how this was performed, see the supplementary appendix in Collins et al. (30). A subset of 50 infants enrolled in the N3RO trial at the Women’s and Children’s Hospital, Adelaide, Australia, consented to participate in this study. Ethics approval for the additional sample collection was obtained from the Human Research Ethics Committee of the Women’s and Children’s Health Network (HREC 2434/12/16). Fecal samples for microbiota profiling were collected from the nappies of each infant between baseline and time of BPD diagnosis (Table 1; Fig. 1). Fecal samples were aseptically transferred to cryotubes and frozen immediately at −80°C for later DNA extraction. A capillary blood sample (0.5 ml) was obtained from infants via heel prick at baseline (induction in the trial) and at 36 weeks postmenstrual age. Blood was collected in anticoagulant tubes containing potassium and sodium EDTA; 0.1 ml was separated into RNase-free microcentrifuge tubes, mixed with 0.3 ml RNAlater (Ambion, Inc.), and stored at −80°C until processing.

### Fecal DNA extraction and 16S rRNA library preparation.

Approximately 0.2 g of stool sample was extracted using the PowerLyzer PowerSoil DNA isolation kit (Mo Bio Laboratories, CA, USA) according to the manufacturer’s instructions, with minor modifications. Samples were eluted in 50 μl of distilled water rather than solution C6. A FastPrep-24 instrument (MP Biomedicals, Santa Ana, USA) was used for sample homogenization, and samples were homogenized with two pulses at 6.5 m/s for 60 s. Total DNA concentrations of all samples were calculated on a Qubit 2.0 fluorometer (Thermo Fisher Scientific, MA, USA) with a high-sensitivity double-stranded DNA (dsDNA) assay kit (Life Technologies Corp., Carlsbad, CA) using 2 μl of extract. Sequence libraries were prepared as previously described by Choo et al. (69). Briefly, the V4 hypervariable region of the 16S rRNA gene was amplified from DNA using the universal bacterial primer pair 515F (5′-TCGTCGGCAGCGTCAGATGTGTATAAGAGACAGGTGCCAGCMGCCGCGGTAA-3′) and 806R (5′-GTCTCGTGGGCTCGGAGATGTGTATAAGAGACAGGGACTACHVGGGTWTCTAAT-3′). Amplicons were then generated, cleaned, indexed, and sequenced according to the Illumina MiSeq 16S metagenomic sequencing library preparation protocol (Illumina, Inc., San Diego, CA, USA). The resulting libraries were then sequenced (2 × 300 bp) on an Illumina MiSeq instrument.

### 16S rRNA gene sequence data analysis.

16S rRNA gene sequences were demultiplexed and imported into QIIME2 (release 2018.8) for processing (70). Sequences were error corrected, and counts of error-corrected reads per sample, which we refer to herein as exact sequence variants (ESVs), were generated with DADA2 version 1.8 (34). A phylogenetic tree of error-corrected sequences was constructed with FastTree (71). Taxonomy was assigned to sequences with the RDP Naive Bayesian Classifier algorithm (72) as implemented in the assignTaxonomy function in QIIME2 (version 1.8.0). All statistical analysis was carried out in R version 3.6.0, with graphing performed using ggplot2 (73). Alpha diversity and Bray-Curtis distances were generated using PhyloSeq version 1.24.2 (74). Principle-coordinate analysis was conducted using the R package Ape version 5.1 (75). Differences in the relative abundances of microbial taxa were assessed using a generalized linear mixed-effects model (GLMM) implemented in the lme4 R package (41). The subject was modelled as a random effect, whereas BPD status, day of life, and DHA treatment were fixed effects. The area under the curve (AUC) was calculated using the AUC function in the DescTools package (76). Coabundant groups (CAGs) of microbes were defined by hierarchical clustering of the Spearman’s rank correlation coefficients of sequence relative abundances. Only those CAGs that were present in at least 20 samples were included in the correlation analysis. R code for all analysis is provided as described in the data availability statement below.

### RNA extraction from blood and library preparation.

RNA extraction and genomic DNA elimination were carried out using the RiboPure kit (Ambion, Inc.) according to the manufacturer’s instructions. Final elution into 10 μl RNase-free water yielded 5 to 20 μg total RNA as determined by analysis of samples using a Bioanalyzer 2100 (Agilent). RNA was transcribed into cDNA in a strand-dependent manner using the Ovation human blood RNA-Seq library systems kit (NuGen Technologies) with 500 μg RNA as the input material. cDNA was sheared into fragments of 200 to 300 bp using a Covaris S220 focused ultrasonicator with empirically determined settings, and the samples were selectively enriched for non-ribosomal RNA and nonglobin sequences via targeted depletion of selected sequences using Insert Dependent Adaptor Cleavage technology. Additional oligonucleotides were also designed and incorporated to deplete the infant-predominant γ-globin, and unique adaptors were incorporated for RNA-Seq multiplexing. The resulting cDNA libraries were visualized on a Bioanalzyer 2100 to confirm the correct size distribution and to determine the cDNA concentration. Libraries were pooled and sequenced using an Illumina HiSeq 2500 machine (1 × 100-bp single-end reads).

### RNA-Seq data analysis.

The quality and number of the reads for each sample were assessed using FastQC version 0.11.4 (77). Read trimming was carried out using Trimmomatic version 0.38 (78) with a window size of 2 and an average quality score of 20. Following this, reads which were <50 nucleotides after trimming were discarded. Reads that passed all quality control steps were then aligned to the human genome (GRCh38 assembly) using HISAT2 version 2.1.0 (79). The gene count matrix was generated with FeatureCounts version 1.5.0-p2 (80) using the union model with Ensembl version 93 annotation. This was then imported into R version 3.5.0 for further analysis. Counts were normalized using the trimmed mean of M values (TMM) method in EdgeR version 3.22.3 (81) prior to multidimensional scaling analysis and differential gene expression analysis (as performed with the glmLRT function). Gene sets were filtered to remove genes with <1 count per million (cpm) in 50% of samples prior to differential expression analysis. Pathway and Gene Ontology (GO) overrepresentation analysis were carried out with InnateDB (82), and cell type expression enrichment was undertaken using CTen (45). HOMER (Hypergeometric Optimization of Motif EnRichment) was used to identify transcription factor binding sites enriched among differentially expressed genes (83).

### Correlating the relative abundances of taxa in the microbiota with gene expression levels in peripheral blood.

Spearman correlation analysis was used to identify associations between the relative abundances of CAGs of ESVs in the microbiota at 1, 2, and 3 months of life and normalized blood gene expression levels at BPD diagnosis in an approach similar to that previously described by the Human Microbiome Project in reference 47. Only genes that were expressed with a cpm of >10 in at least 15 samples were included in the analysis. Samples from both BPD and non-BPD infants from whom both fecal and blood samples were collected were included in the analysis. Prior to correlation analysis, CAG counts were adjusted by fitting each to a mixed-effects model in lme4 where the subject was modelled as a random effect and adjusting for gestational age, sex, and birth mode. The residuals from each model were then used for the correlation analysis in place of the CAG counts. Genes that were correlated with at least one CAG (*P* < 0.05; Spearman’s ρ > 0.3) were identified and analyzed to identify significantly enriched (FDR < 0.01) pathways or Gene Ontology terms. This approach is analogous to methods that have been successfully implemented to detect signatures in genome-wide association data when individual associations do not reach genome-wide significance (48–51). Pathway, GO, or cell type (45) (using the highly expressed, cell-specific HECS gene database) enrichment analysis was performed using a hypergeometric test implemented in R version 3.5.0. The statistical significance of enriched terms was further assessed using a permutation-based approach whereby we randomized the gene labels and repeated the enrichment analysis 1,000 times. Any pathways/GO terms found to be significantly enriched (FDR < 0.01) in any permutation with randomized gene labels were not considered to be significantly associated with CAG relative abundance. The R code for all analysis is provided as described in the data availability statement below.

### Data availability.

16S rRNA gene sequence data have been deposited in the NCBI Sequence Read Archive under BioProject accession number PRJNA517768. RNA-Seq data have been deposited in the Gene Expression Omnibus (GEO) under accession number GSE125873. Count tables, metadata, and R code are available via the N3RO analysis repository in the Lynn Laboratory BitBucket (https://bitbucket.org/lynnlab/n3ro_data_analysis).
