# Supplementary material for: Changes in the Composition of the Gut Microbiota and the Blood Transcriptome in Preterm Infants at Less than 29 Weeks Gestation Diagnosed with Bronchopulmonary Dysplasia
Source: mSystems. 2019 Oct 29;4(5):e00484-19. doi: 10.1128/mSystems.00484-19 (PMC6819732; doi:10.1128/mSystems.00484-19)
Supplement: FIG S1 [file mSystems.00484-19-sf001.pdf]

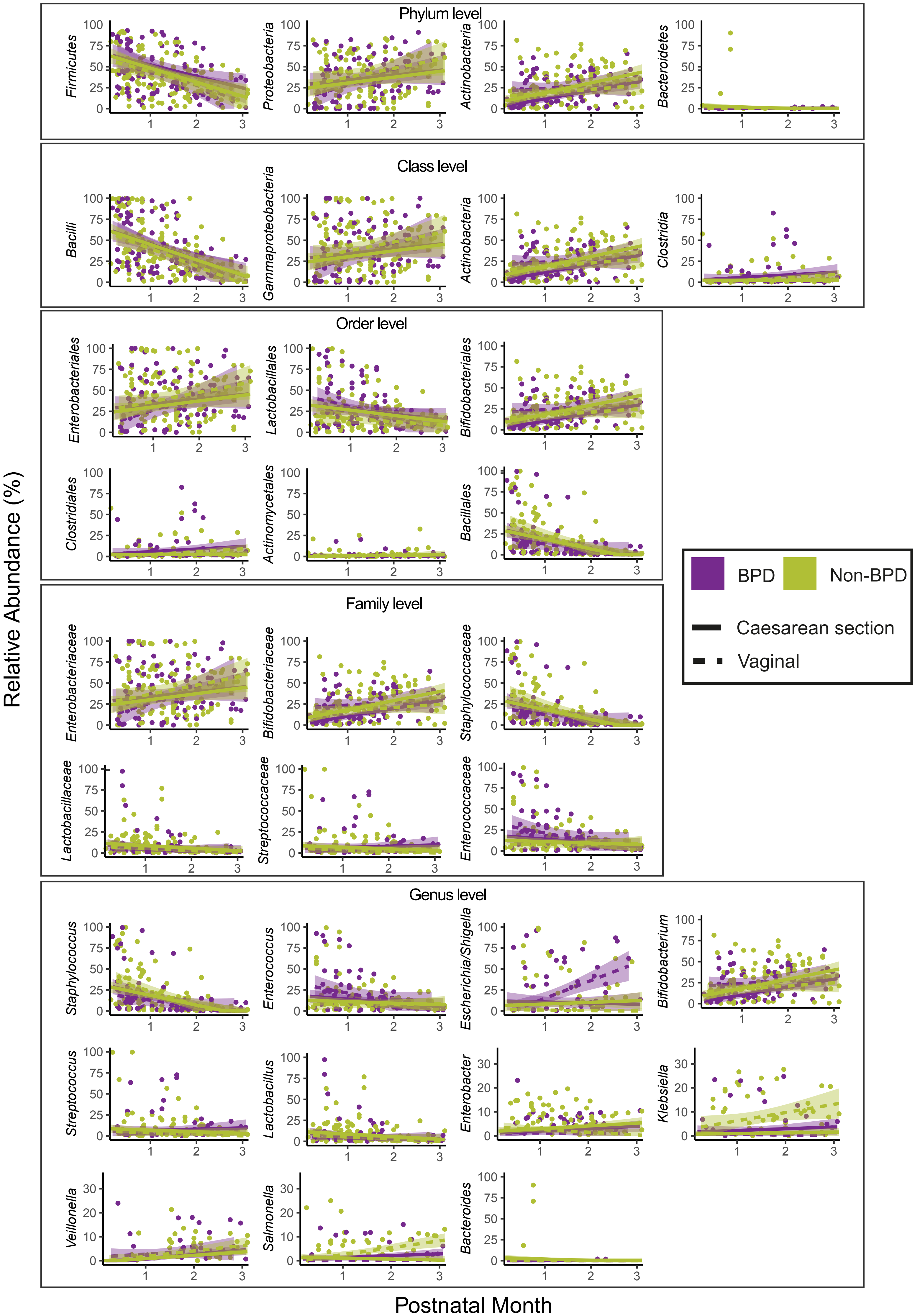

Phylum level
Firmicutes
Proteobacteria
Actinobacteria
Bacteroidetes
Class level
Bacilli
Gammaproteobacteria
Actinobacteria
Clostridia
Order level
Enterobacteriales
Lactobacillales
Bifidobacteriales
Clostridiales
Actinomycetales
Bacillales
Family level
Enterobacteriaceae
Bifidobacteriaceae
Staphylococcaceae
Lactobacillaceae
Streptococcaceae
Enterococcaceae
Genus level
Staphylococcus
Enterococcus
Escherichia/Shigella
Bifidobacterium
Streptococcus
Lactobacillus
Enterobacter
Klebsiella
Veillonella
Salmonella
Bacteroides
BPD
Non-BPD
Caesarean section
Vaginal
Relative Abundance (%)
Postnatal Month
