# Supplementary figures and images for: Changes in the Composition of the Gut Microbiota and the Blood Transcriptome in Preterm Infants at Less than 29 Weeks Gestation Diagnosed with Bronchopulmonary Dysplasia
Source: mSystems. 2019 Oct 29;4(5):e00484-19. doi: 10.1128/mSystems.00484-19 (PMC6819732; doi:10.1128/mSystems.00484-19)

**A**

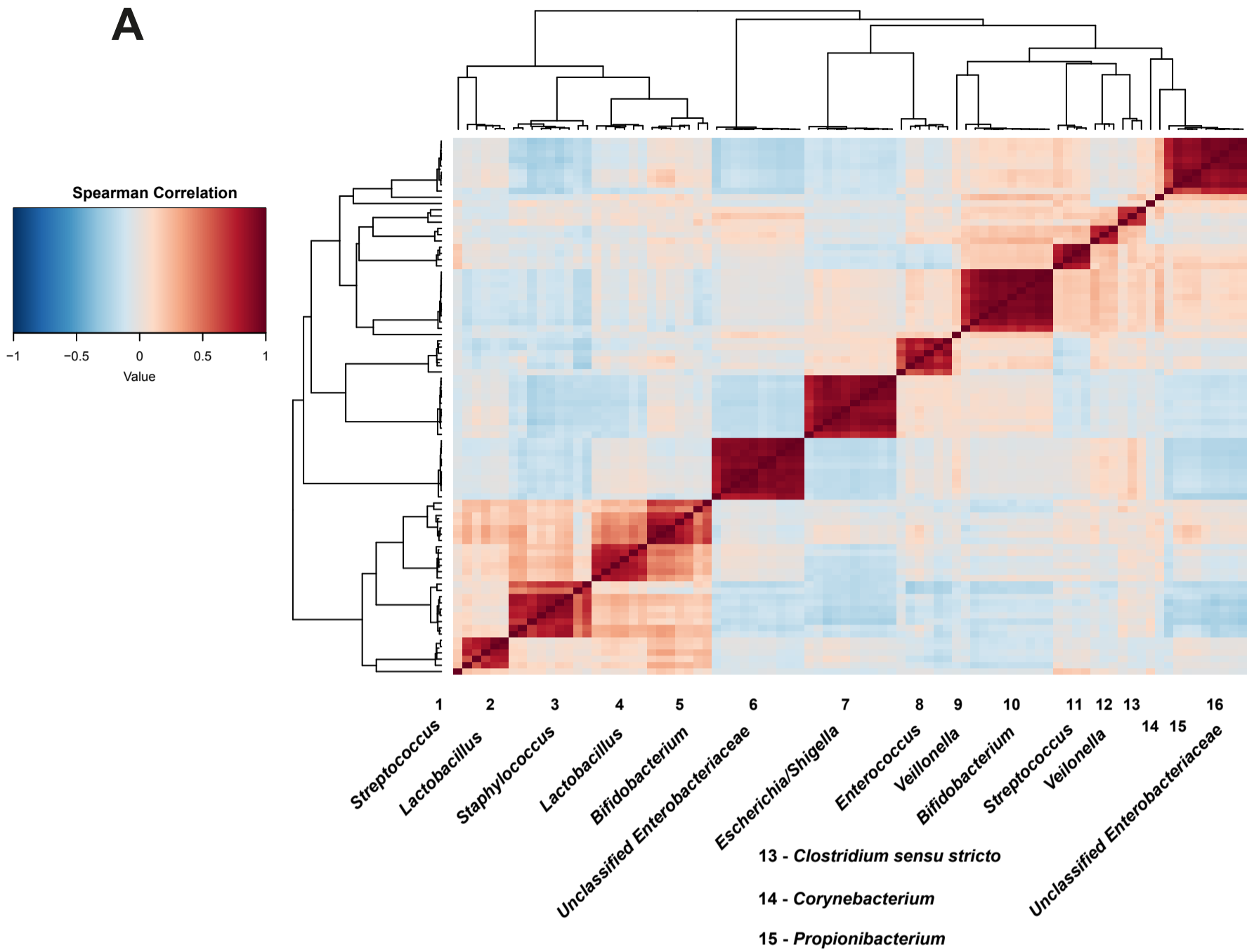

**B**

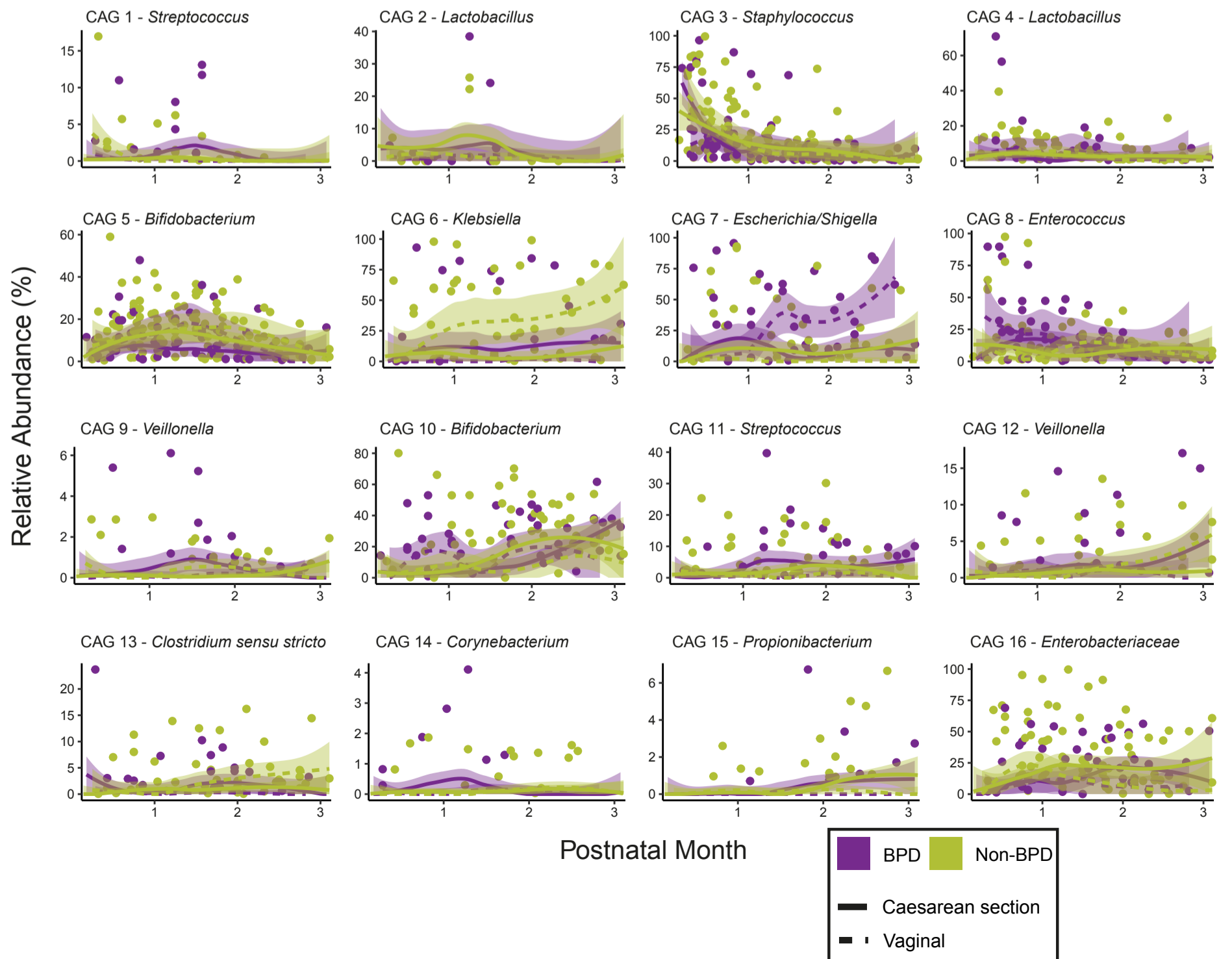

Supplement: FIG S2 [file mSystems.00484-19-sf002.pdf]

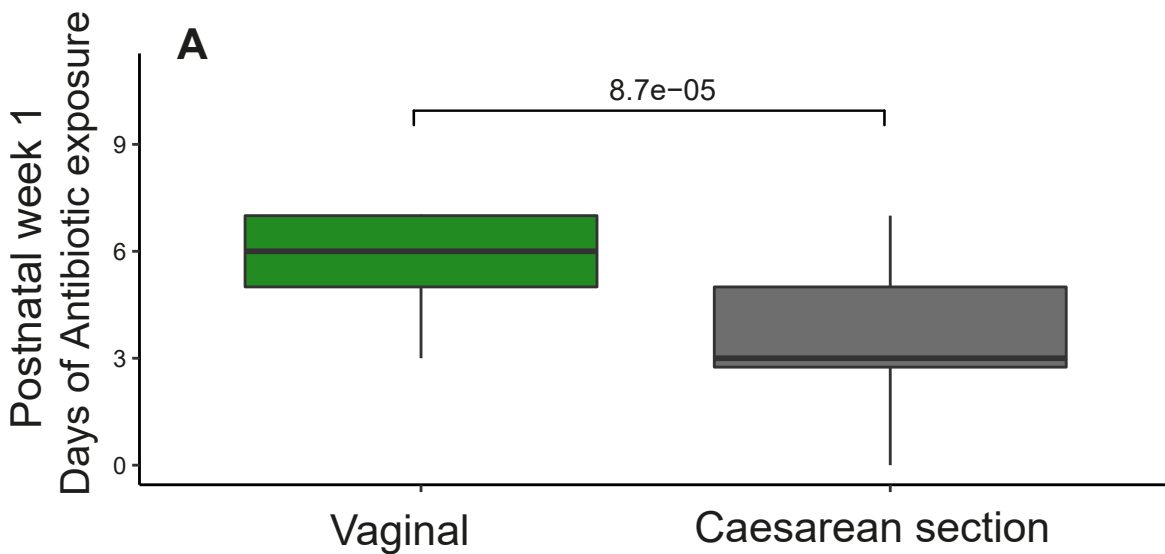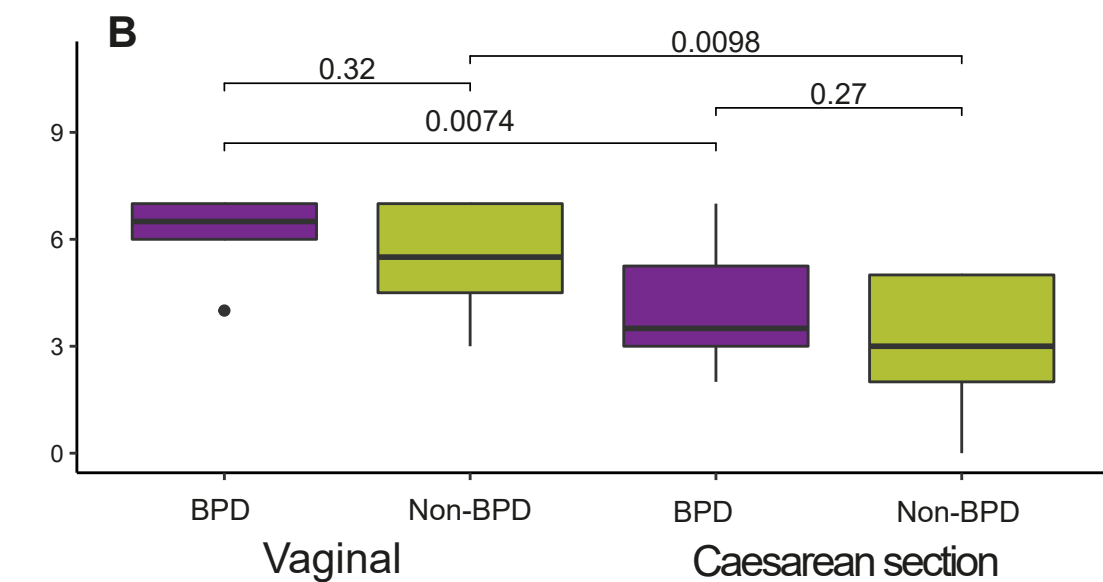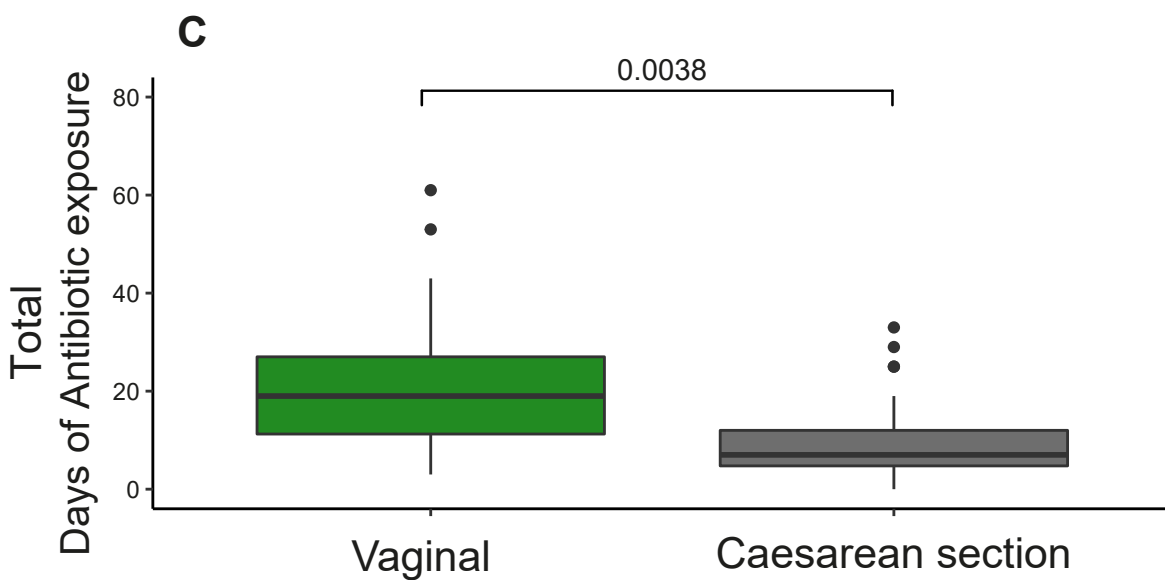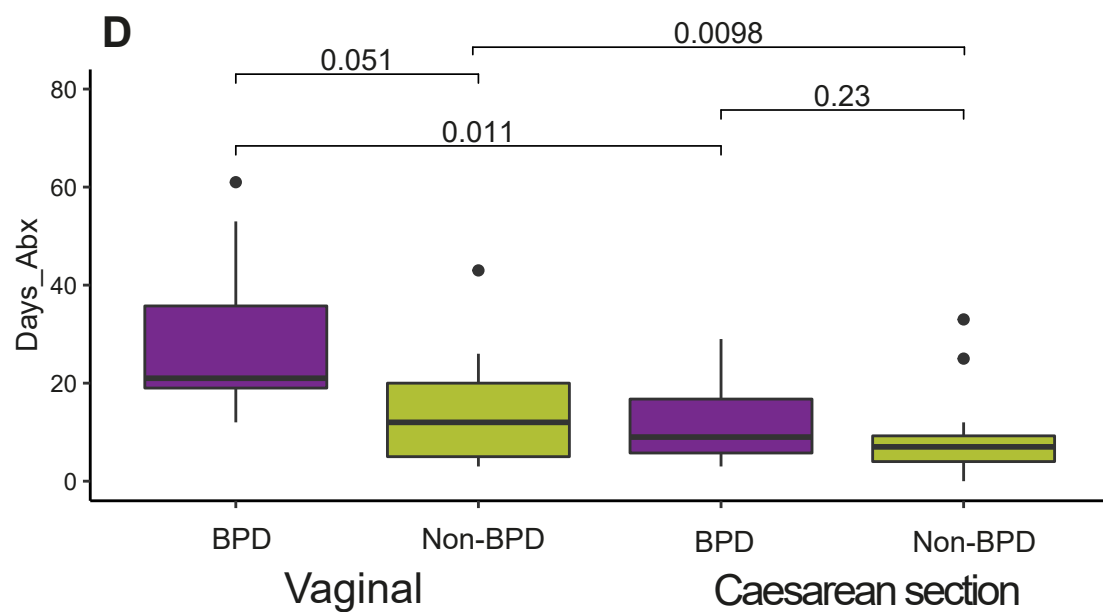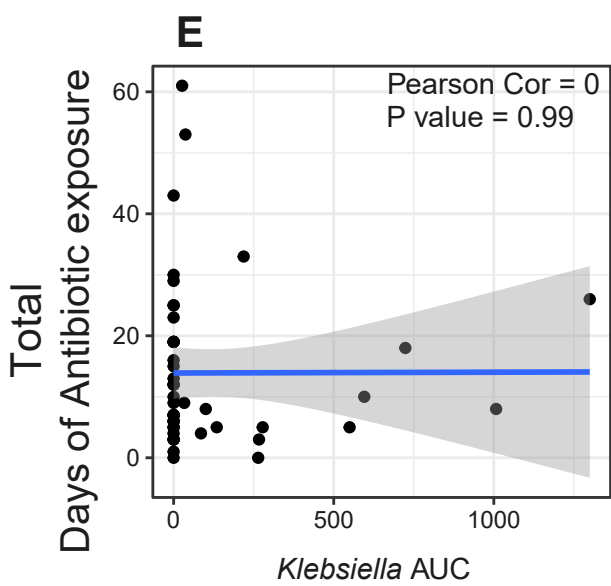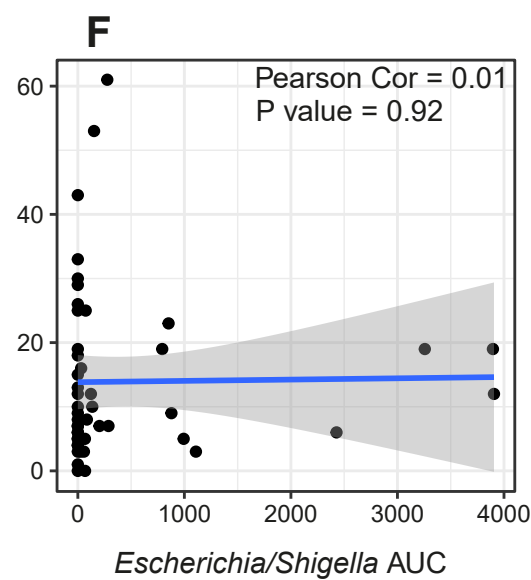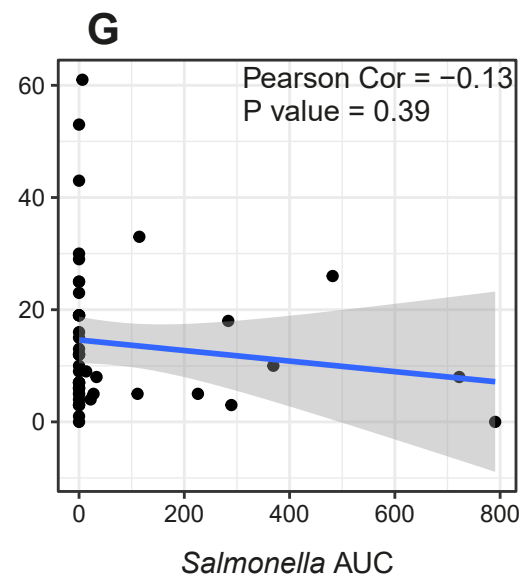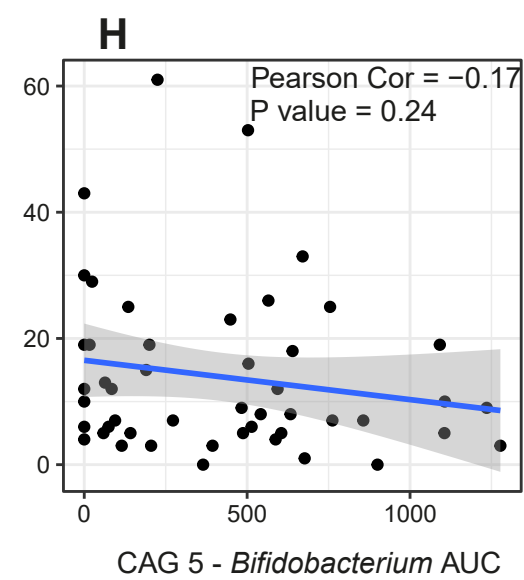

Supplement: FIG S3 [file mSystems.00484-19-sf003.pdf]

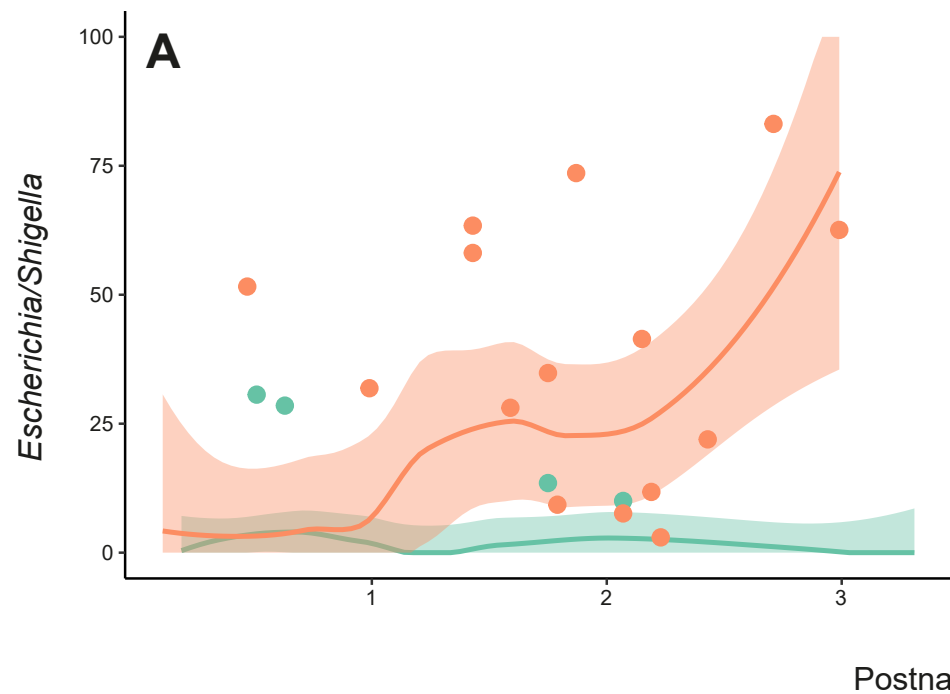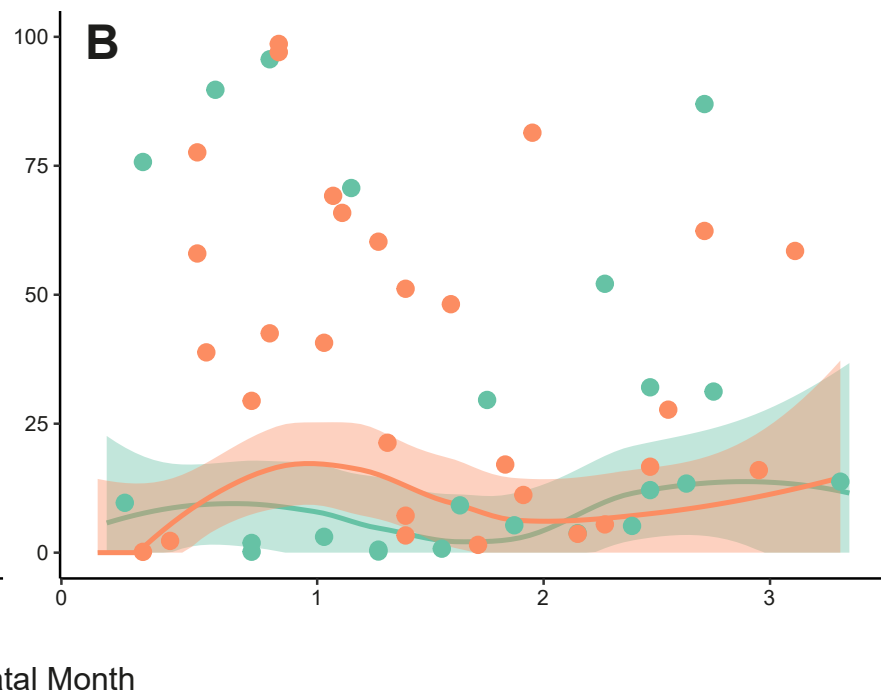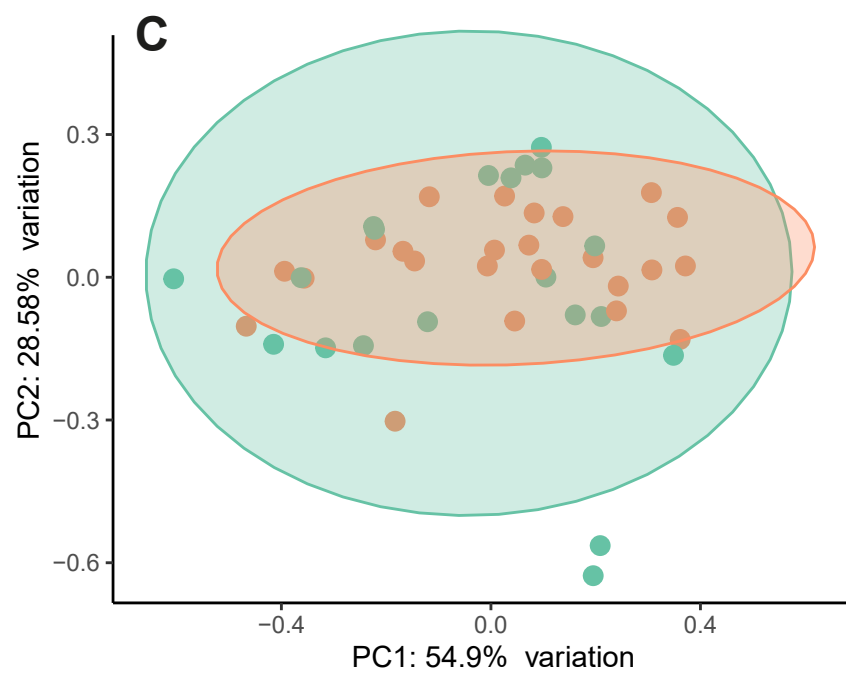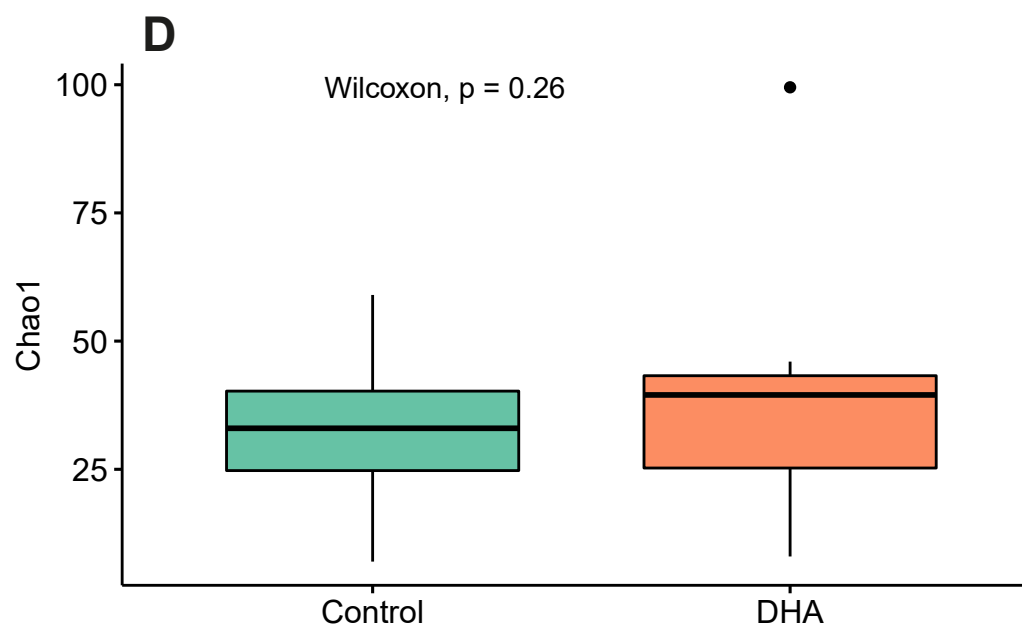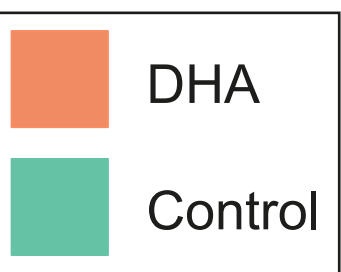

Supplement: FIG S4 [file mSystems.00484-19-sf004.pdf]

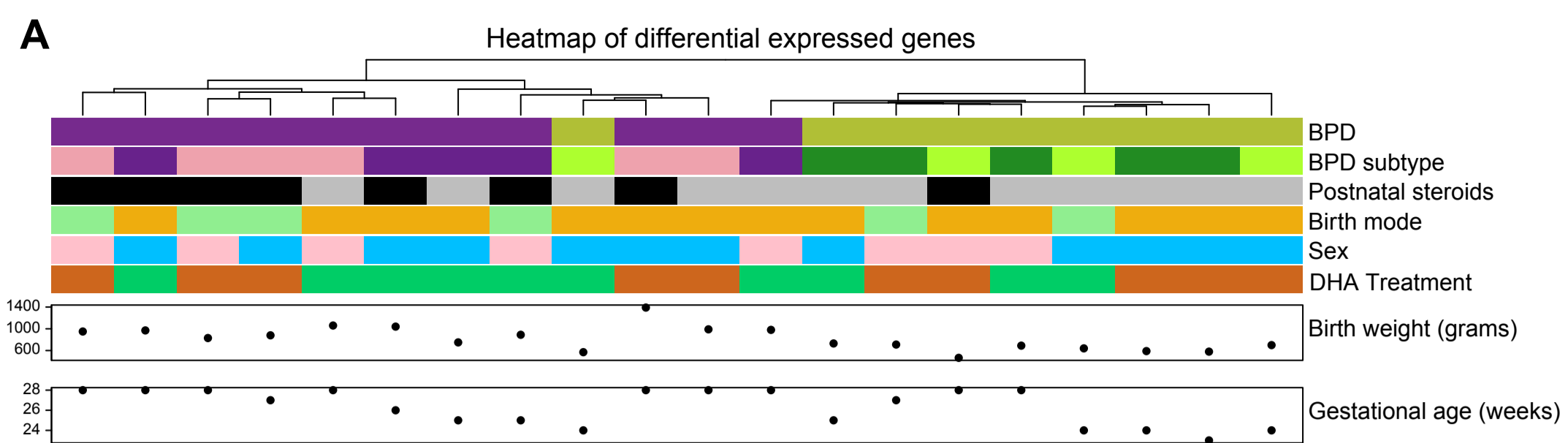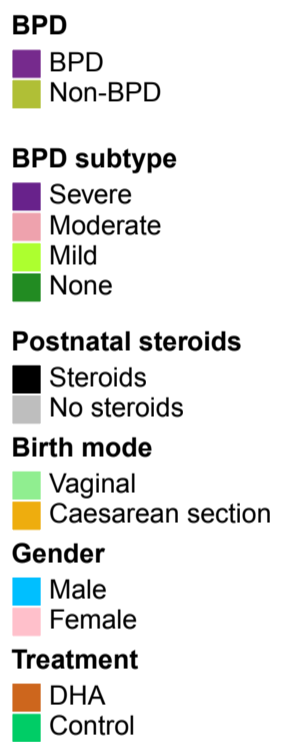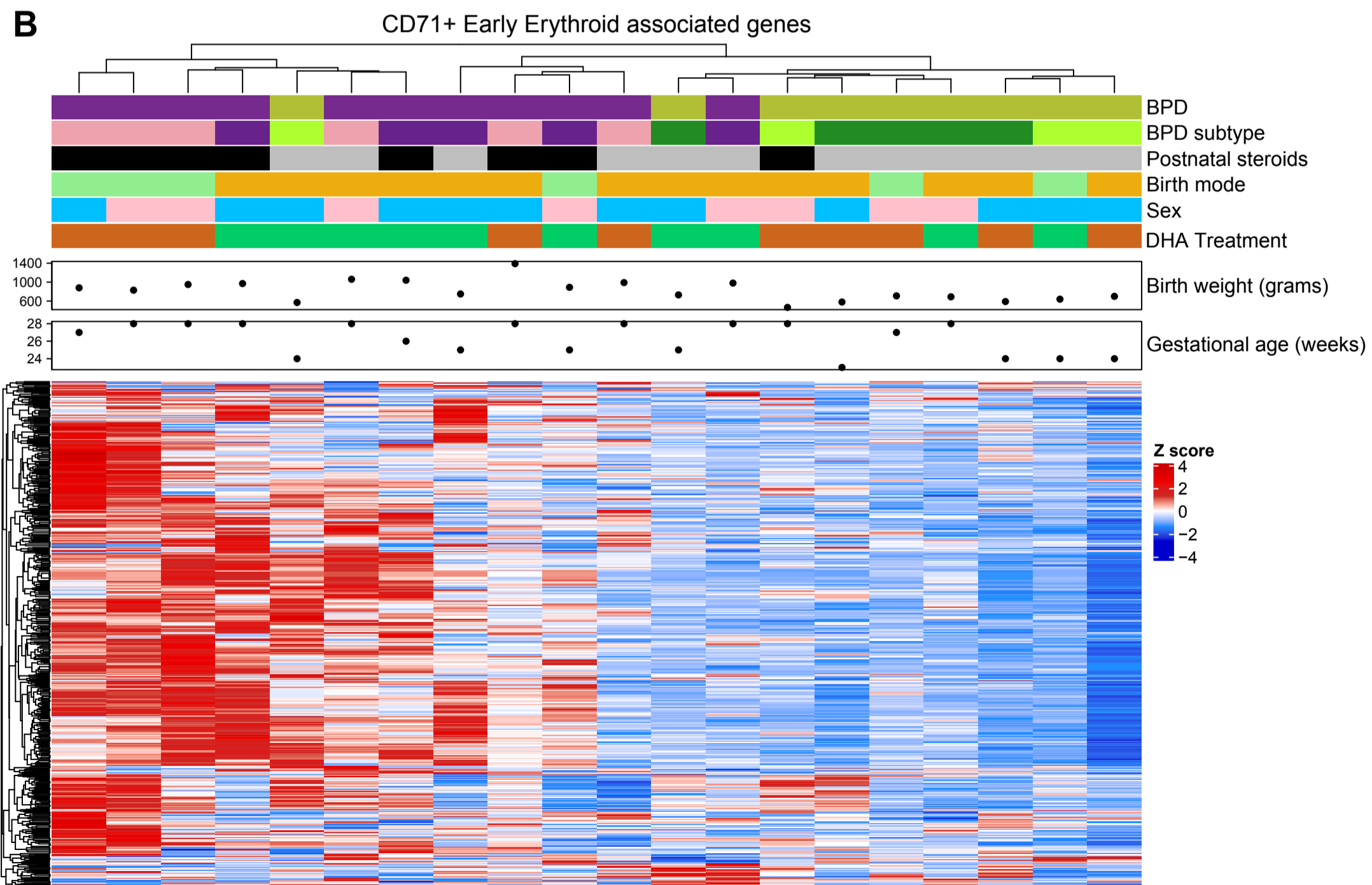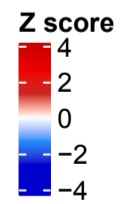

Supplement: FIG S5 [file mSystems.00484-19-sf005.pdf]

A

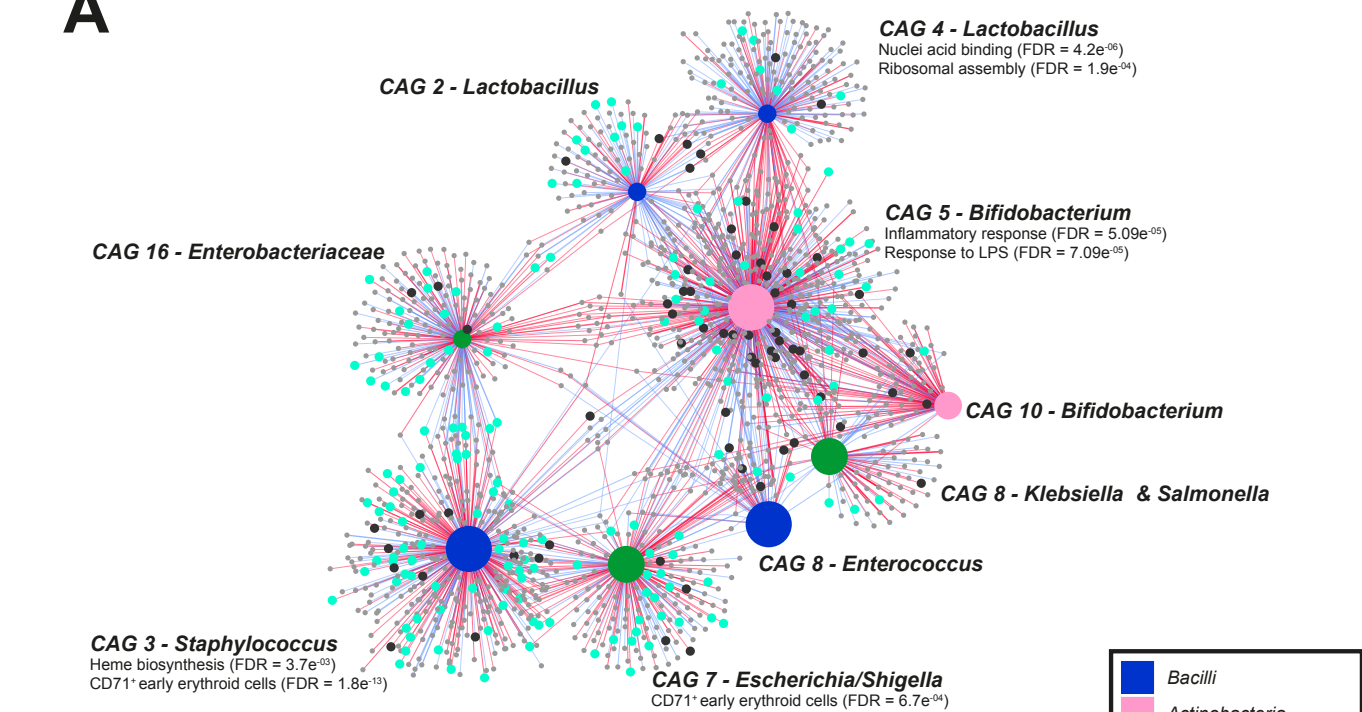

B

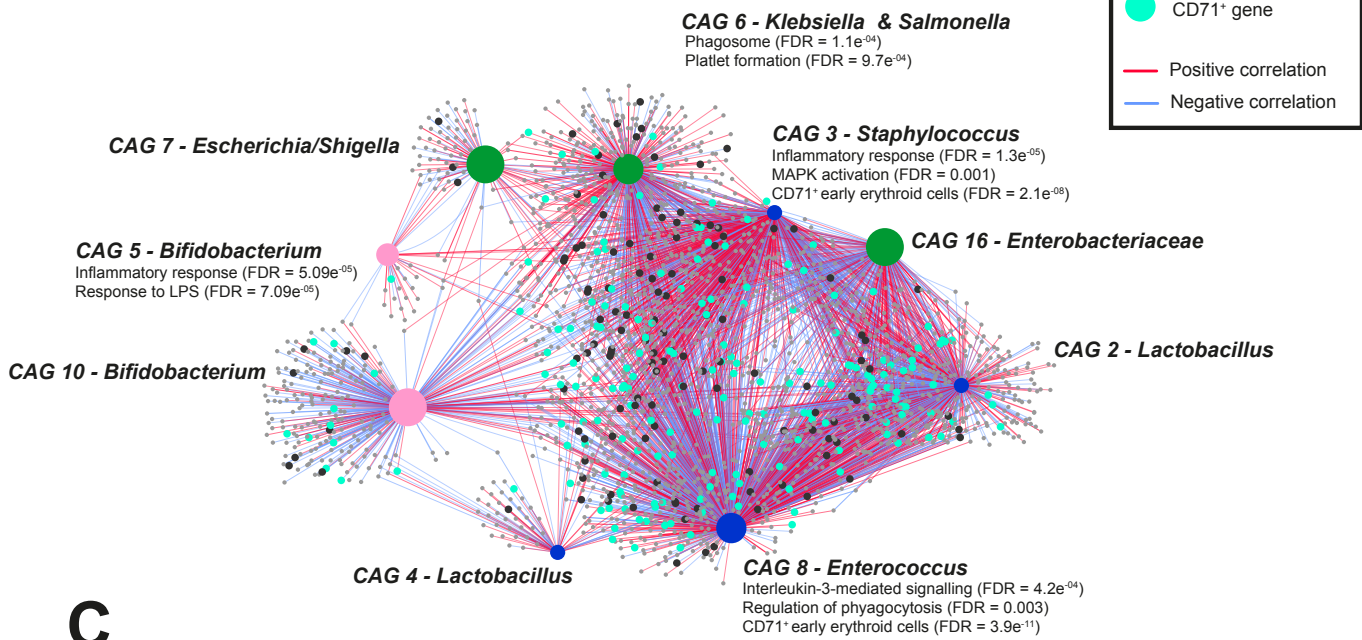

C

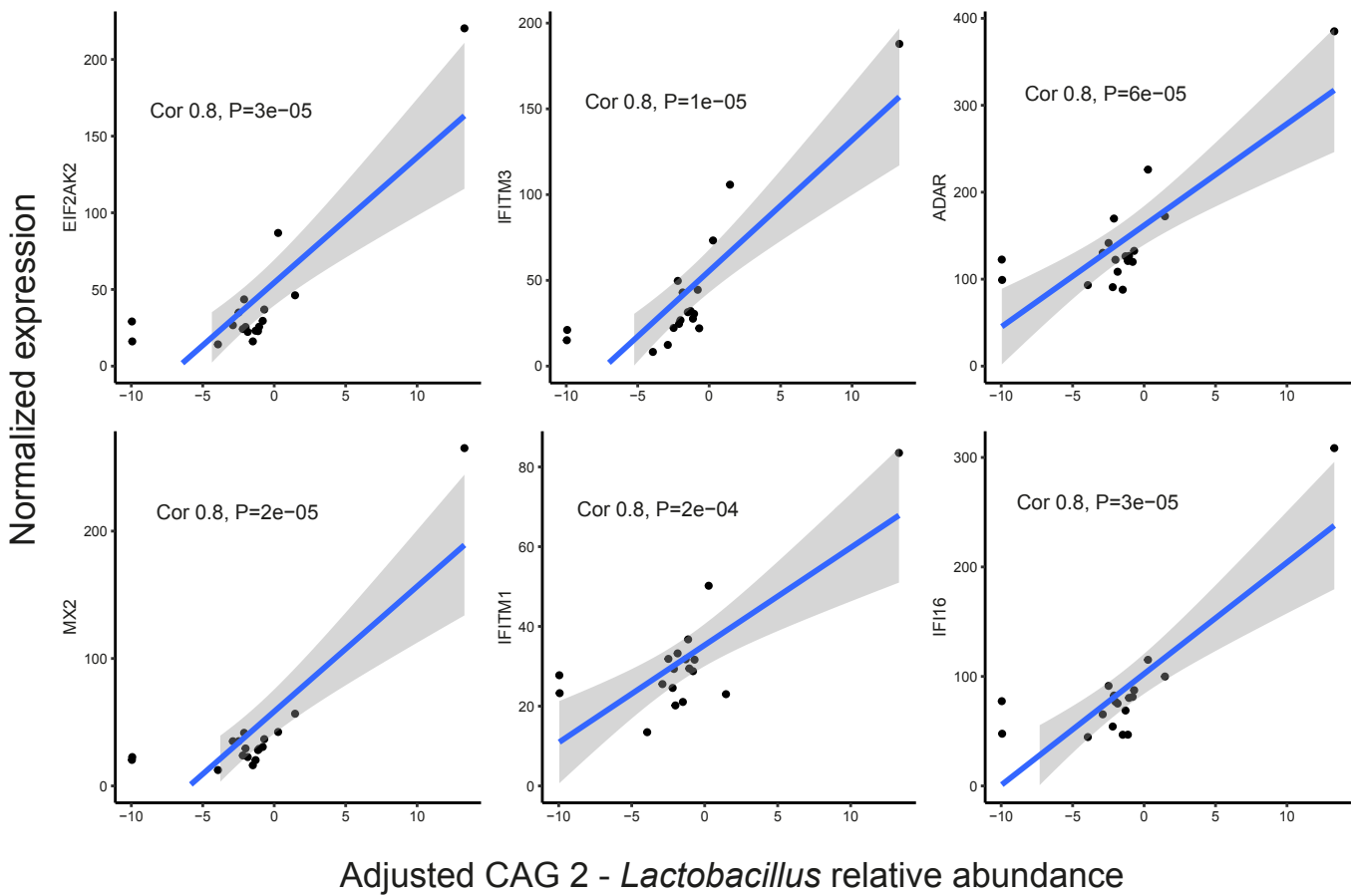

Supplement: FIG S6 [file mSystems.00484-19-sf006.pdf]
